# The complete chloroplast genome of a widespread ornamental shrub in China, *Magnolia figo* (Magnoliaceae)

**DOI:** 10.1080/23802359.2019.1666663

**Published:** 2019-09-17

**Authors:** Yuzhen Zhou, Yan Zheng, Bin Chen, Jinliao Chen, Dong-Hui Peng, Si-Ren Lan, Kai Zhao

**Affiliations:** a Key Laboratory of National Forestry and Grassland Administration for Orchid Conservation and Utilization at College of Landscape Architecture, Ornamental Plant Germplasm Resources Innovation and Engineering Application Research Center, College of Landscape Architecture, Fujian Agriculture and Forestry University, Fuzhou, China;; b College of Life Science, Fujian Normal University, Fuzhou, China

**Keywords:** *Magnolia figo*, chloroplast genome, phylogenetics

## Abstract

Plants in Michelia, presented by *Magnolia figo* DC, are wonderful resources in Magnoliaceae, covering a series of aromatic plants. Despite extensive studies in this family, the *M. figo* complete chloroplast genome and the taxonomical status based on the whole chloroplast sequences remain unclear. Herein, we report the complete chloroplast genome of *M. figo*. The chloroplast genome was 160,113 bp in length, with a large single-copy (LSC) region of 88,113 bp and a small single-copy (SSC) region of 18,797 bp, separated by two inverted repeat (protein-coding) regions of 26,602 bp. A total of 135 CDSs were found, including 129 genes, 85 protein-coding mRNAs, 36 tRNA genes, and eight rRNA genes. The overall GC content was 39.3%, and GC percentages range from 34.3% to 43.2% throughout LSC, IRs, and SSC regions. Phylogenetic analysis showed that *M. figo* is most closely to *Michelia odora* and displayed a relationship that three *Michelia* were nested inside *Magnolia*. This announcement of the complete *M. figo* cp genome sequence could provide valuable information for further breeding, cp genetic modification, and phylogenetic study in Magnoliaceae.

Species in Magnoliaceae occupy important evolutionary positions of the whole plant system; there are meaningful researches to state their taxonomical status in the life tree (Nie et al. 2008). And, several species in this genus are certified as both economically and ornamentally important trees around the world (Shang et al. 2002; Park et al. 2018). *Magnolia figo* (formerly named as *Michelia figo*) is well-recognized as banana shrub in Michelia genus, which contained about 30 species, because of its special attractive flower scent (Holcomb 1999). *Magnolia figo* is an evergreen shrub native to south China and widespread in tropical and subtropical areas around the world (Song and Liu 2019). In recent years, high throughput sequencing tech boosts the taxonomy and functional genome researches; however, magnolias usually possessed huge masses of nuclear genome. It is hard to acquire intact chromosome-level genome information (Chen J et al. 2019). Obtaining a high-quality chloroplast genome (cp) is an ideal approach to excavate some parts of the species characters (Wang et al. 2017). Comparative cp genome of inner or outer species in genus level also provided a new promising method for phylogeny, population dynamics, and species evolution (Li et al. 2019). Thus, we aimed to assemble and characterize the cp genome of *M. figo* to provide a better understanding of the evolution and genetics in genus *Michelia*.

Plant samples were collected and preserved in Fujian Agriculture and Forestry University (location: 26°04′51.3″N 119°14′19.9″E). Total genomic DNA was extracted from fresh leaves by modified CTAB method. The frozen samples including fresh tissues, specimens, and sequenced DNA can be found in the local laboratory of Fujian Agriculture and Forestry University (Voucher specimen accession number: HX-FJ2019-S1, FAFU). Pair-end sequencing library was conducted as PE150 and sequenced by the BGI-500 platform (BGI, Wuhan, China) (Mak et al. 2017). We obtained total about 62.26 Gb clean reads after removing adapters and low-quality reads using fastp software (Chen et al. 2018). Then, the clean reads were mapped to the plant chloroplast reference genome to obtain short pair-end sequences. This recombined reference genome consists of nearby species’ complete chloroplast genome from Genbank, like *Michelia alba* (Accession No. KY204085) and *Michelia odora* (Accession No. NC023239). Filtered reads were then assembled into contigs, scaffolds with low sequence coverages were deleted as noises and ultimately formed the chloroplast circle of *M. figo.* The draft cp genome owning about a 200× average coverage was manually corrected using Bandage v 0.8.1 software. The genome was preliminarily annotated for coding genes and RNA using DOGMA to adjust the starting position. As a result, we established a length of 160,113 bp circle chloroplast genome of *M. figo* with a total GC content of 39.3%. This cp genome is typical includes a length of 88,113 bp large single-copy (LSC) region and 18,797 bp small single-copy (SSC) region, separated by two 26,602 bp inverted repeat (IRs). The four parts manifested an unbalanced GC content. LSC and SSC, 38.0% and 34.3%, are interrupted by two 43.2% GC content IRs from both sides. After assessment of the assembled plastid genome, we annotated the new cp-genome using online softwares, CPGAVAS2 and GeSeq. This cp genome included 135 CDSs, and 129 genes, 36 tRNA, and eight conserved rRNA were found, respectively. The assembled cp genome of *M. figo* and related annotation information can be detected in GenBank with an accession number of MK948432.

The genus *Magnolia* has been found several subgenera divided besides subgenera *Magnolia*, *Yulania Spach*, and *Michelia* L. after preliminary phylogenetic proves revealed genus *Michelia* and subgenus *Yulania* were more closely (Li et al. 2019). Here, we combined the complete cp genomes in genus *Magnolia* to investigate its phylogenetic position, 12 nearby-species complete cp genomes were settled and aligned following HomBlocks pipeline (Bi et al. 2018). Then, RAxML-HPC was used to construct the maximum-likelihood (ML) tree with 1000 bootstrap replicates as shown in Figure 1. As expected, *M. figo* was more closely related to *M. odora* than *M. alba*, forming an independent clade in genus *Magnolia* (Hinsinger and Strijk 2017). This cluster offers a proof that *M. figo* still locates in the subgenus *Michelia* in the level of complete chloroplast genome sequence. We believe the presentation of *M. figo* chloroplast genome helps clarify its evolutionary status in genus Magnolia and provides vital genomic resources for magnolias fast breeding and fundamental researches.

**Figure 1.**
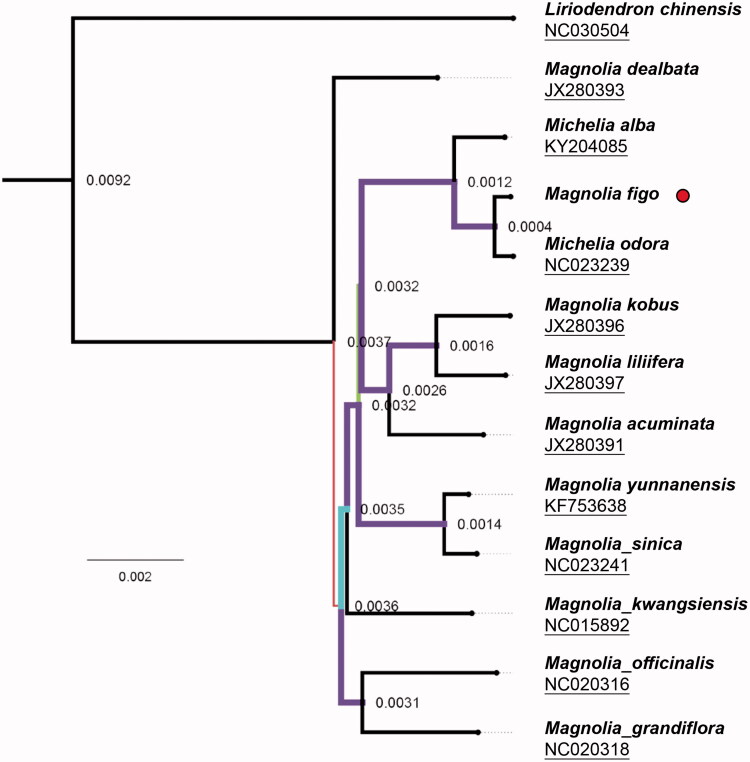
Maximum-likelihood (ML) phylogenetic tree of 13 selected chloroplast sequences in Magnoliaceae with 1000 bootstraps. *Liriodendron chinensis* was treated as the outgroup. *Magnolia figo* were marked with red circle. Genbank accession numbers were listed under its corresponding species.
